# Effect of Continuous Cropping Generations on Each Component Biomass of Poplar Seedlings during Different Growth Periods

**DOI:** 10.1155/2014/618421

**Published:** 2014-10-23

**Authors:** Jiangbao Xia, Shuyong Zhang, Tian Li, Xia Liu, Ronghua Zhang, Guangcan Zhang

**Affiliations:** ^1^Shandong Provincial Key Laboratory of Eco-Environmental Science for Yellow River Delta, Binzhou University, Binzhou 256603, China; ^2^Shandong Province Key Laboratory of Soil Erosion and Ecological Restoration, Forestry College, Shandong Agricultural University, Taian 271018, China; ^3^Forestry College, Nanjing Forestry University, Nanjing, Jiangsu 210037, China

## Abstract

In order to investigate the change rules and response characteristics of growth status on each component of poplar seedling followed by continuous cropping generations and growth period, we clear the biomass distribution pattern of poplar seedling, adapt continuous cropping, and provide theoretical foundation and technical reference on cultivation management of poplar seedling, the first generation, second generation, and third generation continuous cropping poplar seedlings were taken as study objects, and the whole poplar seedling was harvested to measure and analyze the change of each component biomass on different growth period poplar leaves, newly emerging branches, trunks and root system, and so forth. The results showed that the whole biomass of poplar seedling decreased significantly with the leaf area and its ratio increased, and the growth was inhibited obviously. The biomass aboveground was more than that underground. The ratios of leaf biomass and newly emerging branches biomass of first continuous cropping poplar seedling were relatively high. With the continuous cropping generations and growth cycle increasing, poplar seedling had a growth strategy to improve the ratio of root-shoot and root-leaf to adapt the limited soil nutrient of continuous cropping.

## 1. Introduction

Due to the characteristics of fast growth, early lumber, easy to update, and so forth, the poplar is one of the most appropriate management species for industrial timber with short rotation [1]. Poplar is also a good tree species for afforestation and plains in north China, with the characteristics of long planting history, big planting area, and intensive planting measure [2] and had high stand volume per unit area and significant economic and social effect [3]. Long time monoculture and continuous cropping led to the deterioration of soil ecological environment, exhaustion of soil fertility [4], and the decrease of forest growth and productivity [2, 3]. As early as 1869, Germany had found soil fertility exhaustion which was called by a joint name “second effect,” then many countries including Norway, India, France, South Africa, and former Soviet Union, also found the question of plantation soil fertility exhaustion and the decline of productivity, the referred trees included* Picea abies*,* Pinus pinaster*, and* Pinus radiate* [2, 3]. In the 1990s, the research on poplar plantation mainly focused on the mechanism of degradation and the technology of soil fertility holding on continuous cropping poplar forest land [2, 3], the biological characteristics of forest land [5], the rhizosphere effect of continuous cropping poplar plantation [4], and so forth. The research on how the continuous cropping generations led to the change of each component growth was little, and how the continuous cropping effects the distribution of each component biomass of poplar plantation was not clear, resulted in the morphology mechanism of poplar seedling adapting continuous cropping unclear, which limited the cultivation management and pattern allocation of continuous cropping poplar to some extent.

Phytocoenosium biomass, which embodies the combined action of community structure, environment, human activity, and so forth and reflects the matter production, the community structure feature, and growth status of the producer of ecological system, were an important reflection of ecological system productivity [6]. Single tree biomass is one of the main measurement indexes of community structure and function, which can indicate the capacity of plant to fix and accumulate space resource. Each tissue and organ of plant is a unified whole, and the aboveground part has important effect on underground part. The competitive capacity of plant to light resource, underground water, and mineral nutrition was achieved through comparing the biomass distribution ratio of aboveground organic and underground organic [7].

The comprehensive performance of plant growth in all environments is the response of plant biology characteristic. The distribution of plant resource is the centre content on the theoretical research of life history [8] and has important ecological and evolutionary sense, while the division of each component biomass is the response of the limited resource distribution in plant [7]. The variation of biomass distribution pattern, not only is an important way for plant to adapt divergent habitat, but also reflects the change of available resource in environment [9]. The level of each component biomass of plant can reflect the level of photosynthate accumulate in each function part, and the ratio of each component biomass can be used to analyze the change rules of plant biomass distribution patterns [7, 10]. Biomass ratio is the important indicator of plants fitness and reflects trade-off idea [7, 10]. The variation of absolute biomass in plant components reflects the distribution states and optimal distribution pattern of biomass directly, while the relative biomass embodies the dynamic balance of plant physiological metabolism and the circulation of materials, the balance included not only the balance of circulation of materials in plants, but also the balance of plant and environment. Therefore, under the condition of environment variation and stress, the study on the distribution pattern of photosynthetic carbon in plant is more meaningful than that on the accumulation of photosynthate in plant [11]. The growth situation and morphological characteristics of trees during seedling period can preferably reflect the growth potential of plant. The level of each component biomass of plant seedling can comprehensively express the influence of external factor on seedling growth and the adaptive capacity of seedling to external environment [10]. Therefore, the first generation, second generation, and third generation continuous cropping poplar seedlings were taken as study objects, and the whole poplar seedling was harvested to measure and analyze the change of each component biomass of poplar leaves, newly emerging branches, and trunks and root system during different growth period. The purpose was to investigate the response characteristics of growth status on each component of poplar seedling following by continuous cropping generations and growth period, and illuminate the change rules of biomass distribution pattern adapting continuous cropping, thereby to provide theoretical foundation and technical reference on cultivation management of poplar seedling.

## 2. Materials and Methods

### 2.1. Plant Material and Experimental Design

The experimental site is located in Dahesha forest farm, Shan County, Shandong Province, China, which is part of Yellow river delta, and the soil is yellow alluvial soil with light loamy and low organic matter content. It lies at north latitude 34°26′ and east longitude 116°04′. Four-year-old,* Populus deltoids* “I-69” cutting poplar seedlings were selected as the study material and planted in the middle of March 2012. Every tree was selected before explanting to ensure consistent seedling height, diameter at breast height, and growth situations, and the density was 2.0 m × 2.0 m. Under the same site condition, selected sample plot under the condition of first generation, second generation, and third generation continuous cropping, respectively, and the area of each sample plot was 20 m × 20 m with 120 poplar seedlings. In June, August, and the end of October, 30 poplar seedlings were selected every time, and the whole poplar seedling was harvested to measure and analyze the poplar seedling biomass accumulation and distribution. The methods were as follows: dig up the whole seedling and clean it, and four parts were divided, including leaf, one-year-old emerging branches, trunks, and root system. Inactivate enzymes under 105°C, and put them in 80°C to constant weight 30 min latter. Use CID-CI 203 to measure leaf area index (LAI), weigh dry weight of each component and calculate leaf area ratio (LAR), specific leaf area (SLA), leaf mass ratio (LMR), stem mass ratio (SMR), branch mass ratio (BMR), root/shoot ratio (R/S), and root/leaf ratio (RLR).

### 2.2. Statistical Analysis

All statistical analyses were performed with SPSS 16.0 and Excel 2007 for Windows. Repeated measurement analysis of variance (ANOVA) was used and differences were considered significant at *P* < 0.05.

## 3. Results and Discussion

### 3.1. The Biomass of Poplar

#### 3.1.1. Total Biomass

**Figure 1:**
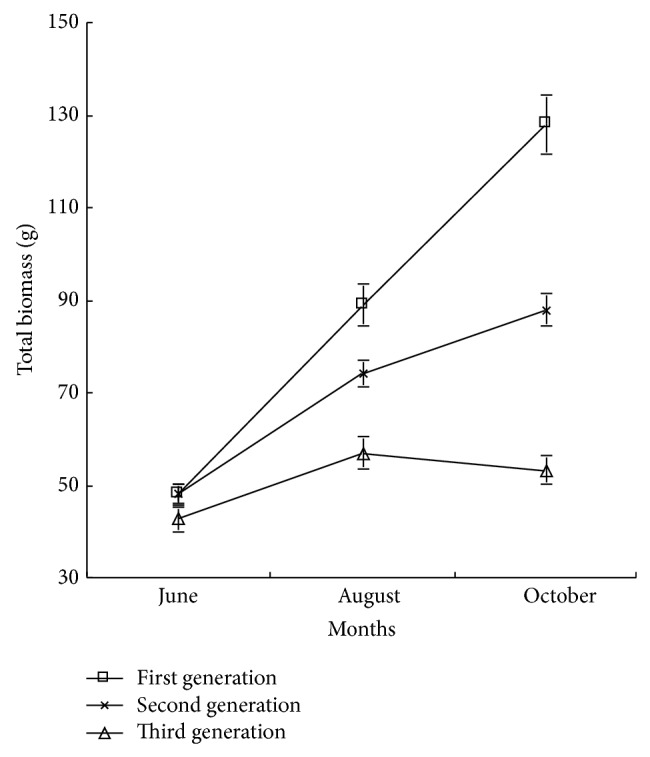
The growth dynamics of total biomass of poplar under different continuous croppings.

Biomass is the best index of forest ecosystem productivity and the most direct expression on the level of forest ecosystem structure and function, which can reflect the comprehensive environment quality of forest ecosystem [6, 10, 11]. Single tree biomass is the main expression on accumulating energy of plant, and the distribution pattern is limited by external environment, plant age, and plant size [12, 13]. As shown in Figure 1, there were no significant difference between the total biomass of first generation and second generation continuous cropping poplar (*P* > 0.05) in the early growth period, but there was significant difference in the middle growth period and the late growth period (*P* < 0.05). With the plant growing, the differences of total biomass among the first generation, second generation, and third generation were gradually enlarged, totally followed by first generation, second generation, and third generation. Seeming from the same generation, the difference of biomass of first generation and second generation was significant (*P* < 0.05) in the middle and the late growth periods, while there were no significant difference in the third generation biomass (*P* > 0.05); namely, the August later, the growth of third generation poplar was slow. In the middle growth period, the total biomass of first generation and second generation increased by 56.1% and 30.1% compared with that of third generation, respectively, while in the late growth period, the total biomass increased by 140.2% and 65.0% respectively. The related research showed that under the condition of continuous cropping, the tree height and the growth of volume of seven-year-old and ten-year-old I-69 poplar and I-72 poplar expressed a down trend with the increasing generation [1]. The stand biomass of the third generation and fourth generation poplar decreased significantly, and the serious spike top, putrid root, and death of whole plant appeared [3], which was similar to the conclusion that with the increasing generation, the total biomass of poplar decreased. In the period of seedling growth, the adjacent plants competed for sunshine, water, and nutrient [14]. Continuous cropping can lead to lack of soil nutrient, while the decrease of soil nutrient was most significant when the continuous cropping just had single poplar species. The above analysis showed that continuous cropping can inhibit the growth of poplar seedling, and the sequence of the biomass of poplar seedling in each growth period was followed by the first generation, the second generation, and the third generation. With the generation and growth cycle increasing, the inhibition influence was more and more significant, which might be related to the exhaustion of soil caused by continuous cropping [1].

#### 3.1.2. Aboveground and Underground Biomass

**Figure 2:**
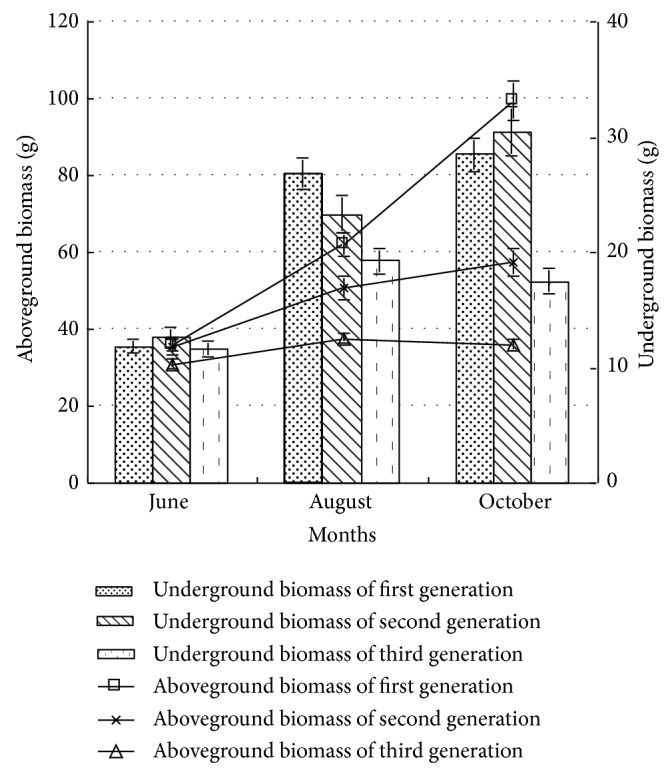
The aboveground and underground biomass of poplar under different continuous croppings.

The plasticity of biomass distribution, which runs through the whole life history of plant and decides the acquiring ability to different resources, has an important ecological significance [15]. Plant made respond to compete recourse through regulating the biomass distribution aboveground and underground, in order to ensure maximizing the limited resource [7]. As shown in Figure 2, the biomass of poplar seedling aboveground was higher than that underground; the change rules of biomass aboveground showed good consistency with the total biomass; namely, with the generation and growth cycle increasing, the growth aboveground was inhibited. In different growth periods, the sequence of the biomass aboveground was followed by the first generation, the second generation, and the third generation. In early growth period, the growth underground had no significant difference among three generations (*P* > 0.05). In middle growth period, the biomass underground had significant difference (*P* < 0.05), and the sequence was followed by the first generation, the second generation, and the third generation. In late growth period, the biomass underground had significant difference (*P* < 0.05), with the sequence followed by the second generation, the first generation, and the third generation, and the first generation poplar grew slowly. In late growth period, the biomass aboveground and underground of first generation and second generation poplar was 2.78 and 1.61 times, 1.63 and 1.74 times that of third generation, respectively. The analysis showed that the growth aboveground and underground of poplar was inhibited by continuous cropping, but the influence aboveground of continuous cropping was higher than that of underground, indicating that the inhibition aboveground was mainly caused by continuous cropping. In early growth period, the influence of continuous cropping on the growth was less, but with the growth cycle increasing, the inhibition was more obvious. In middle and late growth periods, the influence of continuous cropping on the growth underground of first generation and second generation poplars had no significant difference (*P* > 0.05) but had significant difference (*P* < 0.05) aboveground, while the growth of third generation was obviously inhibited by continuous cropping.

### 3.2. The SLA and LAR of Poplar Seedling

**Figure 3:**
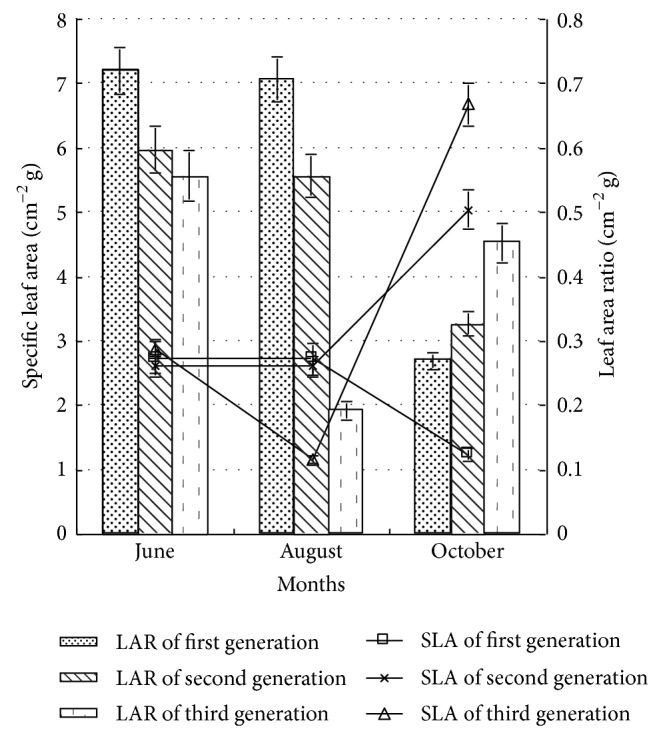
The specific leaf area and leaf area ratio of poplar under different continuous croppings.

SLA and LAR are the important indexes to the regulation of the function of plant. With the adversity stress decreasing, the leaf assimilation rate in unit area was higher, meanwhile major LAR and SLA ensured higher light resource capture area, and both interactions ensured high biomass accumulation [11, 13–15]. As shown in Figure 3, in early growth period, there was no significant difference (*P* > 0.05) among the SLA of first generation, second generation, and third generation. In middle growth period, the SLA of first generation and second generation poplars was obviously higher than that of third generation poplar and was 2.31 and 2.21 times that of third generation poplar, respectively. In late growth period, the SLA of third generation poplar increased significantly and was 5.40 times that of the first generation poplar and 1.33 times that of the second generation poplar, respectively. To the same generation, in middle growth period, there was no significant difference (*P* > 0.05) between the SLA of first generation and second generation poplars. With the growth cycle increasing, the SLA of third generation poplar decreased first and then increased. According to the optimal distribution theory, plant could use the most restrictive resource to acquire component of organic under stress condition, which would get the first priority configuration [16]. Therefore, the SLA of the third generation poplar in late growth period showed increasing trend, in order to get higher light resource capture area sufficiently, indicating that the photosynthetic rate of plant unit mass leaf and the relative growth rate of leaf expressed increasing trend [7].

As shown in Figure 3, in early and middle growth periods, the sequence of LAR was the first generation, the second generation, and the third generation, but in early growth period, there was no significant difference (*P* > 0.05) between that of the second generation and the third generation. In late growth period, the LAR of third generation increased by 40.35% and 27.79%, respectively, comparing with that of first generation and second generation. The LAR of the third generation increased, while that of first generation decreased, which was mainly related to the biomass increasing of the first generation, and that decreasing of the third generation. To the same generation, the LAR of first generation and second generation poplar changed rarely in early and middle growth periods, while decreasing significantly in late growth period. The fluctuation of LAR of third generation was larger and lowest in middle growth period.

The above analysis showed that the influence of first generation and second generation on the SLA was rarely in early and middle growth periods. With the growth cycle increasing, the influence of continuous cropping on the SLA and LAR increased; namely, with the generation increasing, the leaf biomass of poplar seedling decreased, and the SLA and LAR increased, indicating that poplar showed some morphological adaptation strategy to maintain self-survival or biomass accumulation, in accordance with the optimal distribution theory [16].

### 3.3. The Distribution Ratio of Each Component Biomass of Poplar Seedling

The adjustment to the biomass distribution pattern, which was able to let plant balance the relation of resource acquisition and using better, had important significant for plant to maintain regular physiological activity under stress condition. The response of the biomass distribution pattern to environment was the key factor for plant resource acquisition, competition, and reproductive capacity, which was also the key index to reflect the plant competitive capacity, and affected the conformity of organic. The influence of individual development on the biomass of plant organs, which was different to different species and could reflect the sequence change of priority selection on growth, reproduction, defense, and so forth, in the whole growth and development stages, generally was decided by genotype [17] but closely related to heterogeneity habitat [18].

#### 3.3.1. The Ratios of Aboveground Biomass and Underground Biomass

**Figure 4:**
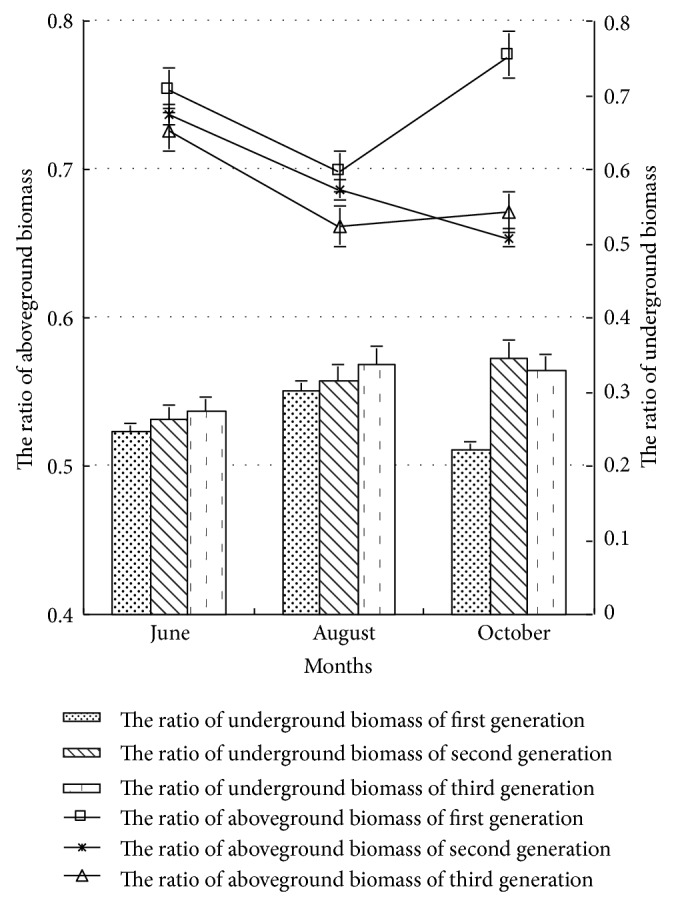
The ratio of aboveground and underground biomass of poplar under different continuous croppings.

The ratios of biomass aboveground and underground could reflect the relative materiality on competition aboveground and underground, as well as reflecting the competitive capacity of plant to environmental resources, such as light, water, and mineral nutrition [7]. As shown in Figure 4, the ratio of biomass aboveground of poplar seedling was higher than that of underground in different continuous generation and growth stages, and the ratios of biomass aboveground and underground showed opposite change rules with the continuous generation and growth cycle increasing. In early and middle growth periods, the ratio of biomass aboveground of first, second, and third generation poplar had significant difference (*P* < 0.05), and the sequence was the first generation, the second generation, and the third generation. In late growth period, the ratio of biomass aboveground of the second and third generation poplars decreased significantly, and that of first generation poplar increased significantly. The ratio of biomass aboveground of the first generation poplar increased by 18.88% and 15.78%, respectively, comparing with that of the second and third generations. In the early and middle growth periods, the ratio of biomass underground of first and second generation poplars had no significant difference (*P* > 0.05), and the sequence was third generation, second generation, and first generation. In late growth period, the ratio of biomass underground of second and third generation poplars was significantly higher than that of first generation (*P* < 0.05) and was 1.55 times and 1.48 times that of the first generation, respectively. For the same generation poplar, the ratio of biomass aboveground of poplar showed decrease trend, but that of biomass underground showed increase trend in middle growth period. Analysis showed that with the generations and growth cycle increasing, the ratio of biomass underground of poplar decreased significantly; while in late growth period, the ratio of biomass aboveground of first generation poplar was obviously higher than that of the second and third generations. The ratio of biomass underground of the second and third generations influenced by continuous cropping was rare, but with the growth cycle increasing, the ratio of biomass underground increased, while that of first generation decreased.

#### 3.3.2. The Ratio of Main Component Biomass

The ratio of each component biomass of plant to total biomass was the result of interaction of self-hereditary character and external environment [10]. The biomass distribution was the key driving factor for plant to acquire net carbon, and the distribution on stem, leaf, and root had direct influence on plant future growth and reproduction [11]. Poorter and Nagel pointed out [19] that not all plants had changeless biomass distribution pattern, which depended on species type, individual development, and habitat environment. The optimal distribution theory predicted that the response of plant to environment was achieved through adjusting the biomass distribution of each organic, in order to maximize acquiring limitation resources, such as light, nutrition, and water [7]. According to the optimal distribution theory and function balance hypothesis, plant should increase the distribution of organics by acquiring limitation resources and reduce the distribution of organics by acquiring nonrestriction resources [20, 21].

**Figure 5:**
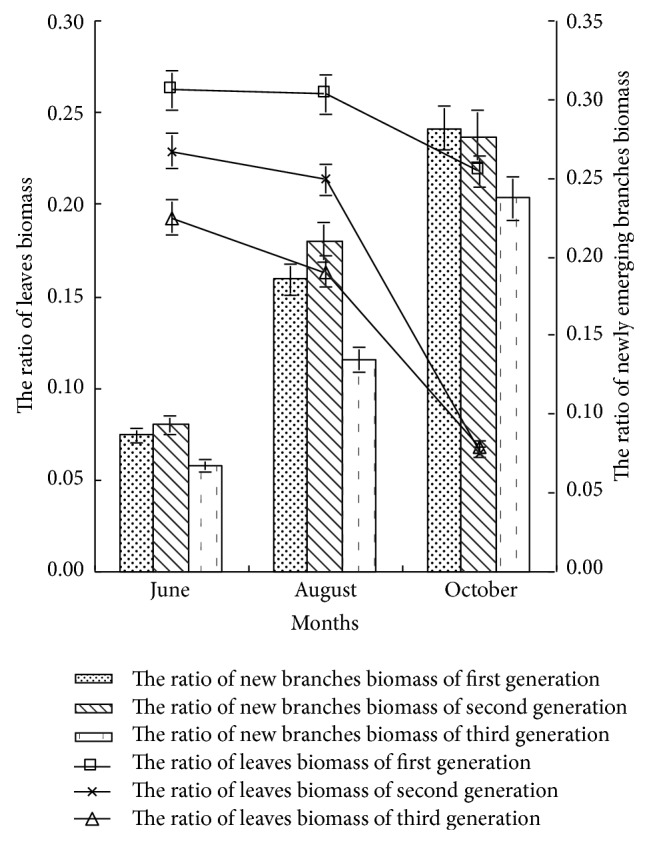
The ratio of leaves and newly emerging branches biomass of poplar under different continuous croppings.

*(1) The Ratio of Leaves Biomass.* As shown in Figure 5, in early and middle growth periods, the ratio of leaf biomass had significant difference (*P* < 0.05) with the sequence of first generation, second generation, and third generation. In late growth period, the ratio of leaf biomass of first generation was obviously higher than that of the second and third generations, and was 3.35 times and 3.21 times that of the second and third generations, respectively, indicating that in the first generation continuous cropping with the soil nutrient sufficient, poplar seedling tended to distribute more organic to leaves, in order to satisfy the demand of seedling to immobilization nutrient through photosynthesis. The rate of leaf biomass of the same generation poplar had no significant difference (*P* > 0.05) in the early and middle growth periods, and that decreased in the late growth period, especially for the second and third generation poplars, indicating that with the generation and growth cycle increasing, the proportion of leaf biomass decreased.

*(2) The Ratio of Newly Emerging Branches Biomass.* As shown in Figure 5, the influence of continuous cropping on the ratio of newly emerging branches biomass of first and second generation was little, but the ratio of third generation decreased significantly. In the late growth period, the ratio of newly emerging branches biomass of the first and second generation poplars increased by 18.99% and 16.88%, comparing with that of the third generation, respectively, indicating that the poplar seedling of the first and second generation had the characteristics of enlarging stems and leaves biomass, increasing the photosynthesis area, and improving the accumulation of substance accumulation, to ensure the demand of seedling growth. With the growth cycle increasing, the ratio of newly emerging branches biomass increased significantly under different continuous cropping conditions. Analysis showed that the third generation continuous cropping obviously inhibited the newly emerging branches growth: with the growth cycle increasing, the inhibited effect of continuous cropping on newly emerging branches growth increased.

**Figure 6:**
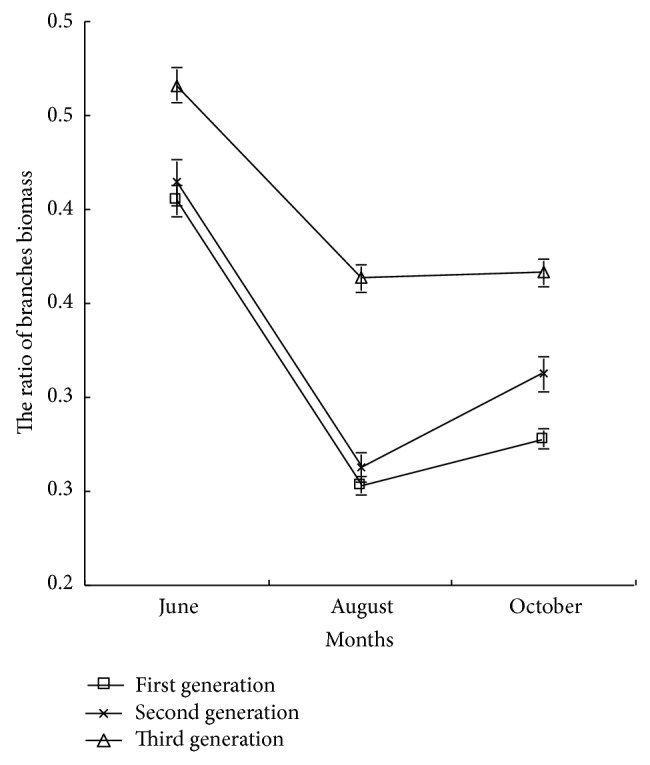
The ratio of branches biomass of poplar under different continuous croppings.

*(3) The Ratio of Branches Biomass.* As shown in Figure 6, the ratio of branches biomass of poplar was followed by third generation, second generation, and first generation, but the difference of first generation and second generation was not significant (*P* > 0.05), and the ratio of branches biomass of third generation was significantly higher than that of first and second generations. In the early growth period, the ratio of branches biomass of the second and third generations increased by 2.48% and 15.53%, respectively, and in the late growth period, increased by 12.23% and 32.01%, respectively, comparing with that of first generation. With the growth cycle increasing, the ratio of branches biomass of poplar decreased rapidly first and then increased. Analysis showed that the growth of poplar branches was slow comparing with other organics, and the growth of plant height had no difference in short continuous cropping; thus the ratio of branches of third generation was higher, further indicating that the growth of leaves and newly emerging branches of third generation was slow.

**Figure 7:**
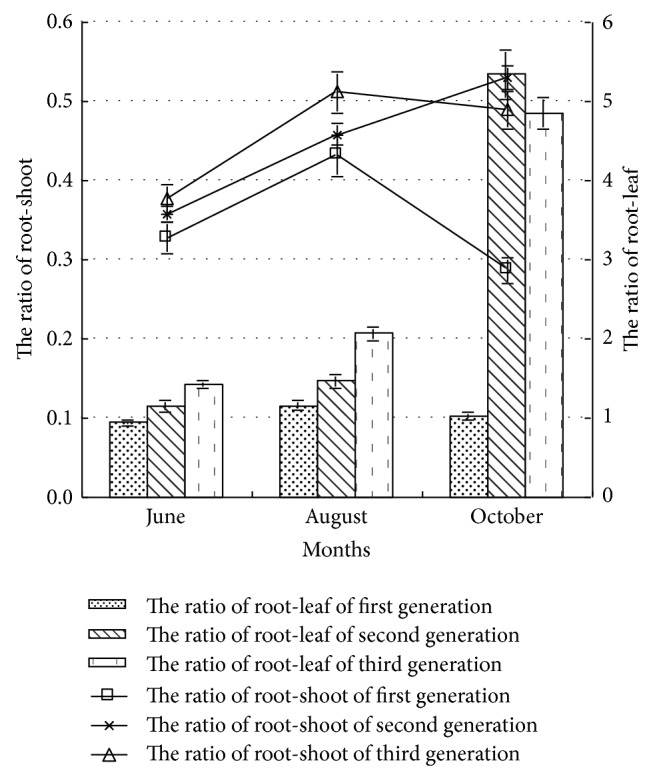
The ratio of root-shoot and root-leaf of poplar under different continuous croppings.

*(4) The Ratio of Root-Shoot and Root-Leaf.* The plant root-shoot ratio, which affected the ecotype of plant individual and the capture strategy to slight resource, and reflected the balance aboveground and underground well, and the function of assimilation and balance of plant to water and nutrient, was one of the main indexes in the analysis of biomass distribution [10]. As shown in Figure 7, in the early and middle growth periods, the root-shoot ratio of poplar was followed by first generation, second generation, and third generation, indicating that the competitive capacity of third generation to soil water and nutrient was strong [10], in order to adapt the poor and severe environment due to continuous cropping. The root-shoot ratio of first generation poplar was the lowest, indicating that the poplar seedling could use major sunlight energy and had high production capacity under first generation continuous cropping condition [7]. In the late growth period, the plant root-shoot ratio of second generation was highest with 0.53, while that of first generation decreased obviously with 0.29, the reason was probably that under first generation condition, in the growth process, the poplar seedling used light resource by enhancing the distribution of tern and leaf, resulting in the plant root-shoot ratio decreasing. To the same generation continuous cropping poplar, with the growth cycle increasing, the root-shoot ratio of first generation increased first and then decreased, while that of the second generation poplar increased all the time. Analysis showed that in order to adjust the soil fertility exhaustion due to continuous cropping, the root system of poplar seedling showed obvious plasticity to assimilate more nutrients. In the early growth period, the influence of continuous cropping on the biomass aboveground was bigger than that on the root system. With the generation increasing, the inhibition effect on the biomass aboveground was higher than that on the growth of root system. Continuous cropping led to the biomass aboveground decreasing, and root-shoot ratio increasing, which was in accordance with the optimal distribution theory. When the plant growth was inhibited by the lack material assimilation by root, the growth of root would show relative advantage, and would lead to the root-shoot ratio increasing [7, 22], which could make the plant under stress condition to increase the most limited resources or organics be distributed optimally [21, 23]. This was similar to the conclusion that the whole biomass and biomass aboveground decreased, but the distribution ratio of photosynthetic production to root and plant root-shoot ratio gradually increased under adversity conditions [22, 24–26].

Root-leaf ratio is the important index to reflect the balance between water absorption and water loss of plant. As shown in Figure 7, in the early and middle growth periods, the root-leaf ratio of poplar seedling was followed by third generation, second generation, and first generation. In late growth period, the root-leaf ratio of second generation poplar was 5.34, while the root-leaf ratio of first generation poplar was 1.02. With the growth cycle increasing, the variation of root-leaf ratio of first generation poplar was rare, while that of second and third generation all showed increased trend, indicating that the poplar seedling distributed more biomass to root, in order to relief the lack of nutrients caused by generation cropping and gain more nutrients from soil, which was in accordance with the optimal distribution theory [24, 27]. In the process of plant growth, plant adjusted the biomass distribution to adapt the change of nutrient condition, when the nutrient was lacking, plant distributed more biomass to roots, and the root-leaf ratio increased; when the nutrient was sufficient, plant distributed more biomass to leaf, and the ratio of leaf biomass increased [26], which was similar to the rules of root system and leaves growth of continuous cropping poplar in the study.

## 4. Conclusions

With the generation and growth cycle increasing, the total biomass of poplar significantly decreased, and the inhibition effect of growth was significant. The growth aboveground of continuous cropping poplar was significantly higher than that underground, indicating that continuous cropping led to slow growth of plant, increment decline, and significant lack of growth potential.

With the generation increasing, both the SLA and LAR of poplar seedling increased, and with the growth cycle increasing, the SLA and LAR of third generation poplar seedling increased significantly. The ratio of biomass aboveground was higher than that underground under continuous cropping condition. With the generation and growth cycle increasing, except in late growth period, the ratio of biomass aboveground increased significantly and the ratio of biomass underground decreased significantly, and the ratio of biomass aboveground of second generation and third generation decreased significantly, while the ratio of biomass underground increased significantly.

The ratio of leaves biomass and newly emerging branches biomass of first generation poplar was higher, and poplar seedling distributed more biomass to the leaves growth and branches growth, which could improve the photosynthetic yield and satisfy the demand of the maximum growth potential of poplar. But with the generation and growth cycle increasing, the ratio of leaves biomass and newly emerging branches biomass decreased significantly. With the growth cycle increasing, the ratio of branches biomass decreased first and then increased in different growth stages with the sequence of third generation, second generation, and first generation.

With the generation and growth cycle increasing, the ratio of poplar root-shoot and root-leaf increased significantly, and more resources were distributed to root system growth, in order to adapt the degeneration of forest soil ecological environment due to continuous cropping, which was in accordance with the optimal distribution theory. Poplar seedling had the growth strategy to improve the ratio of root-leaf and root-shoot to adapt the limited soil nutrient due to continuous cropping.
